# Polyunsaturated Fatty Acids and Their Potential Therapeutic Role in Cardiovascular System Disorders—A Review

**DOI:** 10.3390/nu10101561

**Published:** 2018-10-21

**Authors:** Ewa Sokoła-Wysoczańska, Tomasz Wysoczański, Jolanta Wagner, Katarzyna Czyż, Robert Bodkowski, Stanisław Lochyński, Bożena Patkowska-Sokoła

**Affiliations:** 1 The Lumina Cordis Foundation, Szymanowskiego Street 2/a, 51-609 Wroclaw, Poland; sokola@libero.it; 2 FLC Pharma Ltd., Wroclaw Technology Park Muchoborska Street 18, 54-424 Wroclaw, Poland; tomasz.wysoczanski@gmail.com (T.W.); jolanta.pekala@flcpharma.com (J.W.); 3 Department of Bioorganic Chemistry, Faculty of Chemistry, University of Technology, Wybrzeze Wyspianskiego Street 27, 50-370 Wroclaw, Poland; stanislaw.lochynski@pwr.edu.pl; 4 Institute of Animal Breeding, Faculty of Biology and Animal Sciences, Wroclaw University of Environmental and Life Sciences, Chelmonskiego Street 38c, 50-001 Wroclaw, Poland; robert.bodkowski@upwr.edu.pl (R.B.); bozena.patkowska-sokola@upwr.edu.pl (B.P.-S.); 5 Institute of Cosmetology, Wroclaw College of Physiotherapy, Kosciuszki 4 Street, 50-038 Wroclaw, Poland

**Keywords:** cardiovascular system, omega-3 fatty acids, alpha-linoleic acid (ALA), eicosapentaenoic acid (EPA), docosahexaenoic acid (DHA), lipids, nutrition

## Abstract

Cardiovascular diseases are described as the leading cause of morbidity and mortality in modern societies. Therefore, the importance of cardiovascular diseases prevention is widely reflected in the increasing number of reports on the topic among the key scientific research efforts of the recent period. The importance of essential fatty acids (EFAs) has been recognized in the fields of cardiac science and cardiac medicine, with the significant effects of various fatty acids having been confirmed by experimental studies. Polyunsaturated fatty acids are considered to be important versatile mediators for improving and maintaining human health over the entire lifespan, however, only the cardiac effect has been extensively documented. Recently, it has been shown that omega-3 fatty acids may play a beneficial role in several human pathologies, such as obesity and diabetes mellitus type 2, and are also associated with a reduced incidence of stroke and atherosclerosis, and decreased incidence of cardiovascular diseases. A reasonable diet and wise supplementation of omega-3 EFAs are essential in the prevention and treatment of cardiovascular diseases prevention and treatment.

## 1. Introduction

Fatty acids are considered to be a fundamental building material for the structural components of cells, tissues, and organs as well as for the synthesis of certain biologically active substances. In scientific and medical fields, omega-3 fatty acids are characterized by multidirectional effects in humans: they present anticoagulant and antihypertensive properties, regulate lipid metabolism, and support central nervous system functioning and eyesight. Omega-3 fatty acids also present a wide scope of anti-inflammatory properties, which makes them efficient agents for use in patients presenting various disorders or inflammation-based health conditions. Some researchers have also suggested that omega-3 fatty acids may have an important role in the prevention of various types of cancer [1,2]. This paper introduces the biological significance and health promoting effect of the omega-3 fatty acids family with great focus on the cardiovascular system and its disorders.

Although there has been great progress in the prevention of cardiovascular diseases (CVDs), circulatory system disorders and heart failures are still the leading cause of death globally. It is well known that proper eating habits, regular physical activity, elimination of smoking, and low body weight are essential factors in cardiovascular risk reducing, but despite this common knowledge, CVDs are still the major cause of death worldwide, causing 17.3 million deaths per year (reported by the World Heart Federation in the Urbanization and Cardiovascular Disease report) [3].

At the beginning of the 20th century, cardiovascular diseases were responsible for nearly 10% of all deaths globally. Now, early in the 21st century, they account for nearly one half of all deaths in the developed world and 25% of all deaths in developing countries. Similar data was presented by the World Health Organization as a result of the Global Burden of Diseases Study, which stated that three out of every ten deaths in Europe in patients younger than 65 years old were due to cardiovascular system diseases [4].

Exposure to certain cardiovascular disease risk factors is highly affected by socioeconomic status and the environment in which an individual lives, which means that the majority of CVDs result from risk factors that may be controlled, treated, or modified, which include high blood pressure, cholesterol, diabetes, tobacco use, lack of physical activity, and overweight/obesity.

Consequently, there is enough evidence to conclude that a healthy diet and lifestyle are our best weapons in the fight against cardiovascular diseases and the most effective means in which to prevent the occurrence of CVDs in society. A case study showed that mortality due to ischaemic heart disease and prevalence of coronary arteriosclerosis were low in Greenlandic Inuit (Greenlanders). Since the first description of the so-called Inuit paradox in the 1970s, the number of studies and publications associating polyunsaturated fatty acids intake with lower risk of cardiac disorders has grown extensively [5,6,7].

In the world of cardiology research, remarkably wide recognition in this matter has been obtained as a result of the trials carried out by GISSI (Gruppo Italiano per la Studio della Sopravvivenza nell’Infarto miocardico)—an Italian group studying survival of acute myocardial infarction (AMI). The GISSI designed and carried out a series of large-scale clinical trials that involved more than 60,000 patients over the last 20 years. One of the research studies—the GISSI Preventzioni trial evaluated the effectiveness of a therapy with omega-3 polyunsaturated fatty acids (PUFAs), vitamin E, and a statin in patients with a previous AMI and demonstrated 20% mortality reduction in patients treated with omega-3 PUFAs. Another study demonstrated that omega-3 ethyl esters have clinically proven benefits in improving post-myocardial infarction (MI) outcomes, such as significant 15% risk reduction for all-cause mortality together with 20% risk reduction for CVDs [8,9]. The in-hospital mortality due to AMI has been reduced by approximately 30% over the last 20 years [10]. According to the current guidelines of the European Society of Cardiology, treatment with omega-3 PUFAs may be considered a new option to add to the shortlist of evidence-based life-prolonging therapies for heart failure [11].

## 2. Results

### 2.1. Classification of Unsaturated Fatty Acids

**Figure 1:**
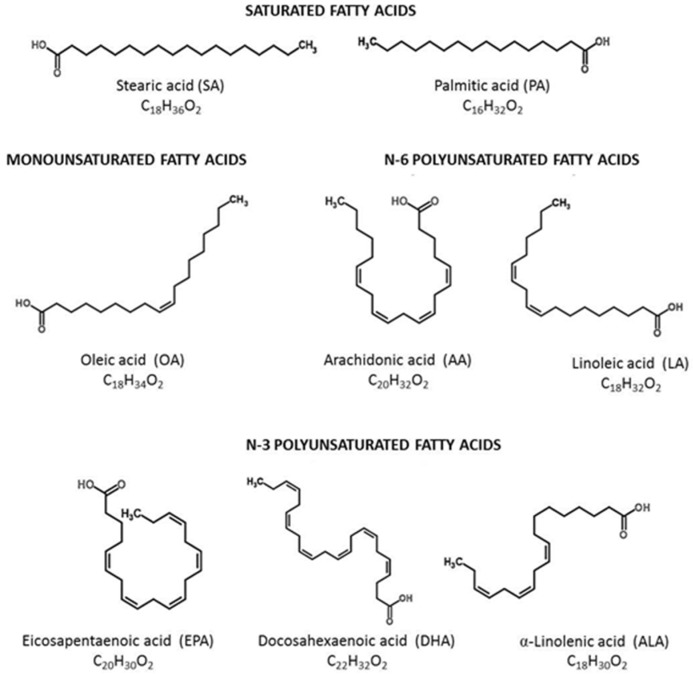
Classification of long-chain fatty acids.

Unsaturated fatty acids (UFAs) are classified as either monounsaturated fatty acids (MUFAs), because they have only one double bond (e.g., omega-7 and -9 fats), or polyunsaturated fatty acids (PUFAs), since they have more than one double bond in their backbone (e.g., omega-3 and -6 acids) [12]. The most important role and significant functions are attributed to PUFAs, which possess a unique subgroup referred to essential fatty acids (EFAs), cannot be synthesized de novo and like vitamins, and need to be delivered with food. They can be further classified in various groups due to their chemical structure. Omega fatty acids are classified according to the location of the first double bond—the difference between them is expressed by the number of omega [13]. Two main compound groups can be distinguished among PUFAs: omega-3 and omega-6 families. The first double bond in the omega-3 family occurs at the third carbon from the methyl end of the chain (hence the name omega-3), and in the case of the omega-6 family, the first double bond occurs at the sixth carbon from the methyl end of the chain (Figure 1).

**Figure 2:**
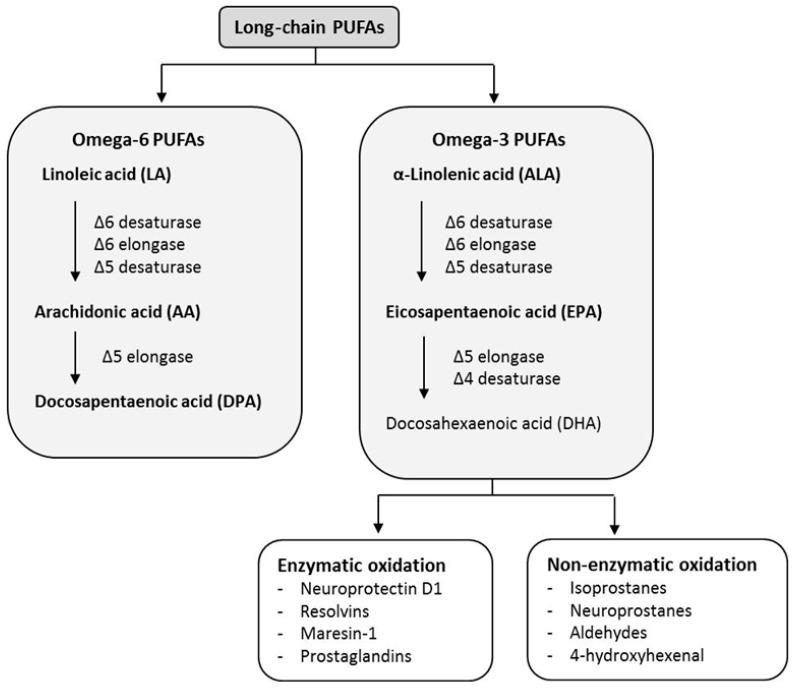
Polyunsaturated fatty acids (PUFAs) subclasses: omega-6 and omega-3.

Alpha-linolenic acid (ALA, 18:3, n-3), eicosapentaenoic acid (EPA, 20:5, n-3), and docosahexaenoic acid (DHA, 22:6, n-3) are the prominent representatives of the omega-3 family (Figure 1). ALA is a precursor of the omega-3 family (Figure 2) and also the only omega-3 that must be derived from the diet, since it cannot be produced by the human body. Regarding food sources, it is found in vegetable oils such as flaxseed (linseed), canola (rapeseed), soybean, and hemp oil, nuts, such as walnuts, as well as in seeds (e.g., chia (*Salvia hispanica*)), dairy products, eggs, and algae [14,15,16,17]. Omega-3 fatty acids can also be found in the meat of free-ranged animals (herbivores and carnivores).

ALA serves as a precursor compound to the synthesis of other omega-3 fatty acids [18]. Although EPA and DHA can also be delivered with food, ALA can be converted to EPA and DHA in the body and therefore has the ability to control their physiological activity. EPA and DHA are commonly found in fish and seafood, especially in fatty fish oils, squid and krill oil, egg oil, and seaweed, hence eicosapentaenoic and docosahexaenoic acids are frequently called “marine omega-3s” [19]. High concentration of these molecules is not fortuitous; their role is to protect these marine organisms from their body fluids solidifying at low temperatures [20].

Omega-6s are represented by the parent compound linoleic acid (LA, 18:2, n-6) (Figure 1). The human body is not capable of producing LA, thus next to omega-3 alpha-linoleic acid, LA constitutes another essential fatty acid that has to be delivered with diet. Once linoleic acid is ingested, it is converted in a few steps into arachidonic acid (AA, 20:4, n-6) (Figure 2). The most significant dietary plant sources of omega-6s are corn, soybean, and sunflower oil, as well as nuts, including coconut together with coconut oil, almonds, pine-nuts, and hazelnuts. Animal origin products that are considered to be good sources of omega-6s include, for example, pork, lard, turkey fat, and butter [21].

It has been established that PUFAs are required for the normal development and functioning of the brain and heart, and also for the equilibrium of all tissues and organs. Studies concerning the nutritional deficiency of omega-3 fatty acids as well as the particular roles of omega-6 and omega-3 have become the focus of numerous research groups around the world [22,23,24,25,26]. Deficit of omega-6 linoleic acid leads to poor growth, fatty liver, skin lesions, and reproductive failure, while the symptoms of omega-3 fatty acids deficiency include reduced vision or abnormal electroretinogram results. Studies in rodents have revealed significant effects of n-3 PUFAs deficiency on learning, memory, cognition, and behavior [27]. The literature reports highlight how the increment of the omega-6/omega-3 ratio corresponds to an increase in the occurrence of pro-inflammatory conditions. It is crucial to maintain a proper balance of omega-3 in our bodies, since an excess of omega-6 leads to low grade chronic systemic inflammation—recognized as the leading cause of the so-called civilizational diseases [28,29,30,31,32].

### 2.2. EFAs Conversion

The concentration of fatty acids in blood reflects both dietary intake and biological processes. When omega-3 and omega-6 PUFAs are consumed, they compete for incorporation into cell membranes in all tissues of the body. Omega-3 and omega-6 fatty acids precursors, i.e., ALA and LA, strive for the same metabolic pathways in the synthesis of longer polyunsaturated fatty acids (such as AA, EPA, and DHA) and for the availability of the same elongase and desaturase enzymes, particularly for Δ6-desaturase. It has been observed that too high an intake of LA would reduce the level of Δ6-desaturase available for the metabolism of ALA [18,32].

The first step of this process involves ALA conversion into EPA, which is an active metabolic product. This is performed through a double bond formation at the sixth and fifth position (by ∆6- and ∆5-desaturase catalysis) and double bond elongation at the sixth position (∆6-elongase). Then, EPA can be metabolized into DHA via ∆5-elongation and ∆4-desaturation [22]. The above process is limited, and the only organs capable of these conversions include the liver, cerebrovascular lumen, and astroglial cells [33,34].

DHA can be further converted into potent novel compounds with anti-inflammatory and organ-protective properties such as the specialized pro-resolving lipid mediators (SPMs), including D- and E-series resolvins, neuroprotectins, and maresins (Figure 2) [35,36,37]. The ability of ALA to be converted into omega-3 long-chain PUFAs may be an important mechanism for maintain adequate EPA and DHA concentrations in cell membranes and thus optimal functioning of the tissues.

Burdge et al., designed a study to estimate the capacity of α-linolenic acid to be converted into omega-3 long-chain polyunsaturated fatty acids [38]. Carbon isotope labelling was used and 13C-ALA was administered orally to six young male subjects who consumed it as part of their usual diet. The results obtained suggested that the liver was the principal site of ALA desaturation and elongation, but the analysis also indicated that approximately 8% of dietary ALA is converted to EPA. There was no significant 13C enrichment of DHA above natural abundance at any of the time points measured over 21 days, thus the percentage of ALA converted to DHA was estimated to be 0–4% [38]. Another study based on radioisotope-labelled ALA application suggested that its conversion to long-chain metabolites is approximately 6% for EPA and 3.8% for DHA in the case of a diet high in saturated fat. However, with a diet rich in n-6 PUFA, this conversion can be reduced by 40–50% [39]. Comparative data have been provided in an analysis conducted by Talahalli et al., on the uptake, tissue deposition, and conversion of ALA to its long-chain metabolites EPA and DHA, compared to EPA + DHA intake [40]. The level of EPA and DHA was measured in rat’s liver, heart, brain, and serum after 60 days of dietary intake of linseed oil, fish oil, and groundnut oil. The obtained results indicated that to maintain a given level of EPA and DHA, required amount of dietary ALA is a few times higher than the combined amount of EPA + DHA (fish oil). Therefore, the efficacy of ALA is lower when compared to applied EPA + DHA in elevating serum and tissue long-chain n-3 PUFA levels [40].

In the omega-6 family, LA is converted to γ-linolenic acid (GLA, 18:3, n-6) due to the activity of Δ6-desaturase enzyme, and then GLA is elongated to form dihomo-GLA (DGLA, 20:3, n-6), which is the precursor of the first series of prostaglandins (PGs). Through Δ5-desaturase action, GLA can also be converted to arachidonic acid (AA), which forms the precursor of the second series of prostaglandins and thromboxanes and the fourth series of leukotrienes.

It is known that the activities of desaturases and elongases involved in the metabolism of EFAs are affected by a number of factors, many of which contribute to reducing the activity of enzymes responsible for ALA and EPA conversion (e.g., smoking, alcohol, stress (adrenaline), deficiencies of certain vitamins or minerals) [41]. Δ6-desaturase activity is inhibited by oncogenic viruses and radiation, while the activity of Δ6 desaturase has been proven to reduce with age [42]. However, in recent years various publications have reported on compounds that could stimulate PUFAs conversion. Co-factors essential for normal ∆6-desaturase activity include pyridoxine, zinc, and magnesium. Δ6-desaturase is activated by insulin, whereas diabetics have reduced Δ6-desaturase activity [43]. On the transcriptional level, peroxisome proliferator-activated receptor-α (PPAR-α) activator WY-14.643 significantly enhanced the transcription of hepatic Δ6-desaturase by more than 500% [44].

Another study based on mice fed with diets containing either a 1.5% fatty acid preparation rich in conjugated linoleic acid (CLA) or rich in LA revealed that dietary CLA concurrently increases the activity and mRNA levels of enzymes involved in fatty acid synthesis and oxidation, as well as PUFAs desaturation in the mouse liver, which appears to be mediated by both the activation of PPAR-α and upregulation of SREBP-1 (sterol regulatory element binding protein-1) [45]. 

Omega-3s and omega-6s present various antagonistic activities. Arachidonic acid undergoes transformation to eicosanoids (leukotrienes, thromboxanes, and the precursor of the first series of prostaglandins), activating the inflammatory and prothrombotic processes, thus facilitating platelet aggregation. AA and EPA also are transformed into their respective hydroxy acids, which in turn are converted into leukotrienes (LTs). Prostaglandins and leukotrienes are highly biologically active, are characterized by pro-inflammatory activity, and are known to be involved in various pathological processes, such as atherosclerosis, bronchial asthma, inflammatory bowel disease, and several other inflammatory conditions.

Hence, most omega-6 fatty acids tend to promote inflammation, whereas the omega-3 fatty acids group has been evidenced to help reduce inflammation. It is worth emphasizing again that some of the most potent inhibitors of desaturases are omega-6 fatty acids, which are present in the modern diet at about 20-fold higher levels than the omega-3s. Nowadays, in addition to saturated fats and trans fatty acid isomers, omega-6s are the most common fat compounds in our modern Western diet, which is rich in vegetable oils, margarine, and deep fried fast food. It has been found that the amount and type of dietary fats in the daily diet and their improper proportions can be involved in the risk of lifestyle diseases, such as obesity, cardiovascular diseases, and cancer, as well as immune system weakening [46,47].

### 2.3. CVD Risk Factors

In its Global Atlas on Cardiovascular Disease Prevention and Control, the World Health Organization (WHO) divided cardiovascular risk factors into modifiable and non-modifiable ones [48]. It reported that most cardiovascular diseases are triggered by risk factors that can be controlled, treated, or modified, such as high blood pressure, lipid disorders, overweight/obesity, tobacco use, lack of physical activity, and diabetes. The WHO together with World Health Federation (WHF) have been raising concerns that in 2008, 9.8% of men and 13.8% of women were obese (with a body mass index (BMI) higher than or equal to 30 kg/m^2^), compared to 4.8% for men and 7.9% for women in 1980. In regard to tobacco users, the WHO estimates that there are currently about 1 billion smokers in the world today. A higher risk of CVDs development was associated with heavy smokers, female smokers and young male smokers [49]. It was emphasized in the report that cardiovascular risk increases with elevated blood glucose values and therefore CVDs account for about 60% of all mortality in people with diabetes.

However, in addition to the variable risk factors, there are also some major CVD factors that cannot be controlled, which include age, gender, and genetic predisposition based on family history. It is well-known that CVDs become increasingly common with age. As a person gets older, the heart undergoes subtle physiologic changes, even in the absence of disease. As the heart muscle undergoes ageing it is often unable to relax completely between beats, and as a result, the pumping chambers become stiffer and may work less efficiently. With respect to the age factor, some studies suggest that men are at greater risk of heart disease than pre-menopausal women. Once past menopause, a woman’s risk is comparable to that of a man. However, the answer for whether premenopausal women are protected from atherosclerotic disease by virtue of their hormonal status still remains inconclusive. The same risk factors for cardiovascular diseases appear to act in women as in men, although the general insufficiency of women presented in a large number of studies means that far less information about the importance of these risk factors is available to us. In light of these data, the risk of stroke is similar for men and women [50,51].

### 2.4. Modern Diet as a Precursor for Inflammation Progression

A healthy diet is a major factor reducing the risk of heart disease. The WHF has reported that comparisons between a diet low in saturated fats, with plenty of fresh fruit and vegetables, and the typical diet of someone living in the developed world show that the former can result in a 73% reduction in the risk of major cardiac events [48]. Awareness regarding the importance of diet in the development and prevention of cardiovascular disease needs to be raised.

A population study has shown that the foods typically consumed in Western diets are heavily laden with fatty acids of the omega-6 family. The current human diet has nothing to do with the diet of our ancestors, and it is generally accepted that hunter-gatherer societies, and other less “Westernized” populations, exhibited superior health markers, such as body composition and physical fitness, when compared to Western populations. Throughout 4–5 million years of hominid evolution, diets were abundant in omega-3 fatty acids from fish and raw meat, but relatively low in omega-6 seed oils. Simopoulos quotes several sources informing that human beings evolved on a diet with a specific proportion of omega-6 to omega-3 fatty acids of approximately 1, whereas nowadays this ratio in Western diets is 15/1–16.7/1 [26,52].

Sources of omega-6 fats include the common vegetable oils used in cooking (corn, safflower, sunflower, etc.), hydrogenated versions of these oils used to make margarine and vegetable shortening, and animal origin food derived from livestock raised on grain, rather than on green pasture. The volume of omega-6 sources in the average diet largely exceeds the volume of omega-3 fatty acids sources—green vegetables, wild ocean fish, flaxseed, walnuts, and animals raised on green vegetation. As a result, Americans usually have omega-6 to omega-3 fatty acids ratios in their tissues on a level of 10:1 to 20:1. With this preponderance of omega-6s in the diet, there is no wonder why people suffer from the consequences of excess omega-6, which are manifested in chronic inflammation, hypertension, as well as an increased blood clotting tendency that elevates the risk for heart attack and stroke.

### 2.5. Lipid Profile

Monitoring and maintaining healthy levels of lipids circulating in our blood stream is important in prevention and early diagnosis of cardiovascular diseases. A lipid profile or lipid panel is a group of blood tests used to evaluate the risk of developing cardiovascular problems or to control an applied treatment.

A lipid profile typically includes total cholesterol, high-density lipoprotein (HDL) cholesterol (the cholesterol in HDL particles), low-density lipoprotein (LDL) cholesterol (the cholesterol combined in LDL particles), and triglycerides (TGs).

HDL cholesterol is considered to be “good” cholesterol, since it helps to remove excess cholesterol from the arteries. HDL particles act as cleaners, carrying LDL cholesterol back to the liver, where it is decomposed and removed from the body. Changes in lipid profile have been associated with cardiovascular diseases due to their key role in the maintenance of the integrity of the cell membrane.

A single LDL particle formed from apolipoproteins B (apoB) is about 220–275 angstroms in diameter, and typically transports 3000 to 6000 fat molecules including cholesterol, phospholipids, and triglycerides (with the amounts of each varying considerably) [53]. Therefore, LDL, the so-called “bad” cholesterol, deposits an excess of cholesterol in the walls of blood vessels, which can contribute to atherosclerosis, ischemic heart disease, or, in some cases, to heart attack. As a quite simple measurement, a lipid profile can provide important information about the progression of diseases.

Since the majority of animal studies were conducted with species that can readily convert ALA to EPA and DHA, it is not easy to isolate the advantages of ALA per se, although there is evidence of ALA affecting vascular function and heart condition [54]. It has been found that dietary flaxseed significantly improves lipid profiles in hyperlipidemic patients and may positively affect cardiovascular risk factors modification [54]. Subsequently, Egert et al., conducted a randomized strictly controlled dietary study to compare the individual effects of dietary ALA, EPA, and DHA on low-density lipoprotein (LDL) and fatty acid composition [55]. Their findings demonstrated how ALA benefited lipoprotein profiles, but in contrast, EPA and DHA led to oxidized LDL formation [55]. Consistent with these results, another study investigated the differential effects of omega-3 PUFAs on metabolic control and vascular reactivity and documented pointed benefits of ALA [56,57]. Goyens et al., evaluated the effects of ALA in comparison to EPA and DHA in a nutritional intervention study [58]. According to their conclusions, in healthy elderly subjects, ALA might affect the levels of LDL-cholesterol and apoB more favorably than EPA/DHA, with the intake level of ALA being of critical importance [58,59].

It is worth mentioning that lipid profile disorders also include abnormalities in triglycerides (TGs) levels in blood plasma. Combined dyslipidemia concerns concurrent unbeneficial changes in various subfractions of lipids, including increased levels of LDL cholesterol and TGs and a reduced level of HDL cholesterol. It was demonstrated in numerous studies that statins, especially at higher doses, are able to reduce blood TGs level, however, it was proved that omega-3 fatty acids are more effective in this case [60]. The recent meta-analysis prepared by Alexander et al. [61] mentioned a prominent effect of omega-3 long-chain PUFAs supplementation in lowering the concentration of serum TGs, and emphasized that an elevated TGslevel is responsible for an increased CVD risk. Thus, the combination therapy of statins and omega-3s may be useful when there is need for the optimization of TGs levels in the event of combined dyslipidemia [62]. It was demonstrated in the study by Weber and Raederstorff [60] that omega-3 fatty acids supplementation may reduce serum TG levels by 20% to 40%, and the authors suggest that this is due to lowered production of very low-density lipoprotein (VLDLs) or an enhanced clearance of chylomicron TGs [63]. It was demonstrated in the study by Davidson et al. [64] that the combined administration of statin (simvastatin—40 mg/d) and omega-3 supplement (465 mg EPA and 375 mg DHA per 1-g capsule) positively affected the blood plasma lipid profile (a decrease in non-HDL-C, VLDL-C, and TG), and this effect was more profound than in the case of statin or omega-3s alone [64]. Also, Micallef and Garg [65] concluded in their study that the potential benefits related to blood lipid parameters such as cholesterol and TGs level can be enhanced using omega-3s as an adjunct to statin therapy.

### 2.6. Atherosclerosis

Healthy arteries are flexible and elastic, but over time, the arteries’ walls can harden and the lumen of blood vessels can become narrower. The direct cause of atherosclerosis is plaque accumulating and building up inside the arteries. Plaque is a miscellany of cholesterol, cells, and debris that creates a bump on the artery wall. It all begins with damage to the endothelium—a thin layer of cells lining the interior surface of blood vessels—that can be caused by risk factors such as high blood pressure, smoking, or high cholesterol, that afterwards leads to plaque formation.

Although atherosclerosis is often considered a heart problem, it can affect any artery in the body, including arteries in the heart, brain, arms, legs, pelvis, and kidneys. As a result, different diseases may develop based on which arteries are affected. 

In the last decades, epidemiological, clinical, and experimental studies have demonstrated that a diet rich in omega-3 plays a central role in atherosclerosis prevention [66,67,68].

Results of various research studies have shown that the suppression of atherosclerosis is associated with reduced levels of serum lipids and antioxidant activity. A recent study hat was designed to evaluate the effects of flaxseed oil containing α-linolenic acid ester of plant sterols (ALA-PS) demonstrated that ALA-PS flaxseed oil synergistically interacted in atherosclerosis ameliorating as well as in optimizing overall lipid levels, contracting inflammation, and lowering oxidative stress [69]. Another animal study suggested that ingestion of oxidized flaxseed oil increases hepatic plasma malondialdehyde (MDA) concentration and is potentially pro-atherogenic. Mice from the control group received fresh flaxseed oil, while the experimental population received the same diet with heated flaxseed oil. The results obtained demonstrated that aortic wall thickness increased and lumen and diameter parameters changed only in the experimental group [70]. Effects of flaxseed on serum lipids in experimental animals have been variable—from no change to spotted reduction. In the study conducted by Prasad, 14 g ALA per day was reported as a threshold dose [71]. The lower dose did not affect inflammatory mediators, but 14 g/d or higher limited and reduced inflammatory markers. The same study also demonstrated that flaxseed can suppress hypercholesterolemic atherosclerosis in a rabbit model.

### 2.7. Cardioprotective Effects via Antithrombotic Activity

Solid experimental evidence of different effects of omega-3 fatty acids on individual components of cardiovascular risk has been described. Multiple research studies revealed that omega-3 long-chain fatty acids may prevent myocardial infarction and arrhythmia, decrease systolic, and diastolic blood pressure and improve vascular function. This effect can be explained by very rapid omega-3 fatty acids incorporation into cell membranes, thus affecting the function of cells and tissues with subsequent impacts on the production of various vasoactive eicosanoids and other mediators [72]. Although the exact mechanisms of the beneficial effect of omega-3s on cardiovascular diseases are multifactorial and remain unclear, a series of omega-3 PUFAs action has been proven and described as mechanisms with major importance for cardiology.

Both anticoagulant and antiplatelet functions of omega-3 PUFAs were explored in the past with inconsistent findings, therefore complementary studies were required [73,74]. Antiplatelet and antithrombotic activity of omega-3 PUFAs has been documented in patients with coronary artery disease (CAD) in an experimental study where PUFAs were distributed independently or in a combined treatment [75,76,77] (e.g., with aspirin and clopidogrel [78,79]). The results obtained may be of significant clinical importance, as they indicated the ability to improve platelet response to clopidogrel by omega-3 PUFA co-administration in coronary artery disease patients treated with angioplasty (percutaneous coronary intervention) who are carriers of the loss-of-function CYP2C19 genetic variant. These findings are also supported by the results of the TRITON-TIMI 38 study subanalysis [80].

Antithrombotic potential was also confirmed in a study based on dietary administration of oil-rich fish (500 g/week for 4 weeks), which resulted in reduced platelet-monocyte aggregation in the study group compared to the control [81]. Additionally, when administered concomitantly with policosanol (10 mg/d)—a mixture of higher aliphatic primary alcohols purified from sugar-cane wax—oil-rich fish were shown to be a safe and effective as a lipid-lowering agent [82,83,84].

A recent study by Mozaffarian and Wu reviewing available evidence for cardiovascular effects of n-3 polyunsaturated fatty acid provides a compelling indication regarding the beneficial effects of omega 3 fatty acids in reducing the risk of cardiac death [85]. Experimental studies confirmed that omega-3 fatty acids may improve cardiac function due to their anticoagulant, anti-triglyceridemic, antihypertensive, hemostatic, and antiarrhythmic properties [46,86,87,88,89].

### 2.8. Antiarrhythmic Properties

Antiarrhythmic properties are of particular interest to researchers. The antiarrhythmic effect of omega-3 fatty acids has been widely described and explained by their effect on the ionic currents and sodium channels in the plasma membrane of cardiomyocytes [90,91,92]. The results of previous studies show that free PUFAs can reduce the membrane electrical excitability of heart cells. Impact on sodium channels causes a shift in the state of dynamic balance inactivation towards hyperpolarized potentials. Consequently, the cardiomyocytes are less susceptible to stimulation. Furthermore, it appears that omega-3 PUFAs are characterized by even higher efficacy in arrhythmia conditions triggered by fresh ischemia (this situation often results in ventricular fibrillation). It was also confirmed in a study involving an animal model. A concentrate of free fish-oil fatty acids in oral administration was tested with respect to its prevention effect on sudden cardiac death in dogs, and the obtained results indicate that purified omega-3 fatty acids could prevent ischemia-induced ventricular fibrillation in the applied dog model [93].

Regarding the suggestion that the cardioprotective effect of fish intake is mainly due to the antiarrhythmic properties of marine n-3 polyunsaturated fatty acids, which modulate ion currents, omega-3 PUFAs of vegetable origin were also examined. Epidemiological studies and dietary trials in humans suggest that α-linolenic acid is a major cardioprotective nutrient [94,95]. The effects of ALA on the specific Kv1.5 channel have also been examined. The results of the studies conducted by Dhein et al. or Guizy et al. indicated that ALA directly blocks atria-specific Kv1.5 channels without modifying their expression or the bilayer order. Together with the indicated influence of EPA and DHA, these effects suggest that the antiarrhythmic potential of diets enriched with plant-derived n-3 PUFAs may partially result from direct effects on cardiac ion channels [96,97].

### 2.9. Myocardial Infarction

Myocardial infarction (MI) or acute myocardial infarction (AMI), commonly known as a heart attack, occurs due to damage to the heart muscle caused by stopped blood flow, which prevents blood from reaching the heart—this refers to STEMI (ST-elevated myocardial infarction) or injured heart muscle caused by major obstruction in blood flow through coronary arteries (NSTEMI—non-ST-elevated myocardial infarction). The MI mechanism often involves the complete blockage of a coronary artery caused by a rupture of an atherosclerotic plaque. Low density lipoprotein (LDL) can contain 70% total plasma cholesterol and—as mentioned previously—is considered the foremost malefactor triggering inflammatory processes and early plaque formation, which later might lead to MI and stroke. It was identified by Assmann and Gotto that reduction in the LDL levels could lower the incidence of coronary heart disease (CHD), including MI, by up to one-third. HDL (the good lipoprotein, which carries only 20% total plasma cholesterol) has also been linked to the rates of coronary events in epidemiological and clinical studies [98]. Although LDL is the lipoprotein most commonly associated with atherosclerosis and cardiovascular risk, other lipoproteins, such as VLDL (very low-density lipoprotein), might also act as atherogenic factors. On the other hand, HDL appears to play a protective role, and high levels of HDL particles are associated with a lower risk of coronary artery disease. The cardioprotective effects of HDL have been attributed to its role in reverse cholesterol transport [99,100]. The results of a clinical study on non-insulin-dependent diabetic individuals consuming a diversified ratio of dietary polyunsaturated to saturated fatty acid (P/S) indicated that fish oil significantly reduced plasma triacylglycerol levels (*p* < 0.05) and increased EPA and DHA content of all lipoprotein lipid classes. Demonty et al., concluded that a modest intake of omega-3 fatty acids, such as could be obtained due to regular fish consumption, would reduce plasma triglyceride levels without affecting LDL or HDL cholesterol levels [101]. It is worth mentioning that an increasing amount of data indicate that to initiate atherosclerosis, LDL has to undergo chemical modification, such as oxidation or glycation, since these particles are more liable to retention within the vessel wall intima [102]. Modified or damaged LDLs or their particular types (e.g., VLDL) have become a subject of interest in regard to their role in CVDs. Thus, when it comes to cholesterol particles, a high LDL level by itself cannot be considered to be the decisive factor responsible for cardiovascular dysfunctions. Current evidence suggests that cardiovascular risk depends on the “quality” rather than only the “quantity” of LDL [103,104].

The Lyon Diet Heart Study found plasma ALA to be associated with an improved prognosis for recurrent myocardial infarction, but it did not find a similar association with long-chain omega-3 fatty acids. A meta-analysis of five prospective studies on ALA suggested that high ALA intake was associated with reduced risk of fatal heart disease (relative risk 0.79, 0.60–1.04) [105]. The average highest level of intake was 2 g per day versus the lowest of 0.8 g per day [105]. Omega-3 fatty acids may provide rapid protective effects in patients with AMI, according to the results of a randomized, placebo-controlled long-term trial. The effects of treatment with fish oil (eicosapentaenoic acid, 1.08 g/day) and mustard oil (α-linolenic acid, 2.9 g/day) were compared for 1 year in the management of patients divided into a fish oil group, a mustard oil group, and placebo patients with suspected AMI. The fish oil and mustard oil groups showed a significant reduction in total cardiac arrhythmias, left ventricular enlargement, and angina pectoris compared to the placebo group [106]. Sun et al., have recently presented how plasmatic long-chain omega-3 PUFAs levels are associated with a lower risk of AMI in Asian population [107]. The case-control study results did not indicate conclusive findings. According to the authors, plasma ALA may be slightly associated with reduced AMI risk, even in individuals with high concentrations of long-chain omega-3s, and this may be partially mediated by lower blood pressure and LDL cholesterol level [107]. Contrary to the pointed importance of the aforementioned finding, the results of the study by Derbali et al., proved that flaxseed oil has a significant effect in heart protection against isoproterenol-induced myocardial infarction. Linum oil pre-co-treatment was reported as an agent preventing almost all induced MI parameters through the beneficial effect of the important ALA fraction [108].

### 2.10. Atrial Fibrillation

An abnormal heart rhythm characterized by irregular, commonly rapid beating, which affects about 2 to 3 per cent of the population in Europe and North America, is another common concomitant disease with coronary artery disease, myocardial infarction, and cardiomyopathy. This condition, i.e., atrial fibrillation (AF), has become one of the most important public health problems over the last 20 years, and is a significant cause of increasing health care costs in Western countries [109].

An epidemiologic approach has been applied in order to evaluate the relationship between plasma ALA levels, dietary ALA consumption, and AF risk. Fretts et al.’s research group investigated subjects who were 65 years or older at study entry and detected no relationship between plasma ALA and AF incident after correcting for age, sex, and a variety of clinical and demographic factors [110]. However, a technical weaknesses of the study included plasma ALA levels measurement only occurring at a single time point [110,111]. It should be mentioned that a similar epidemiologic study has recently reported a beneficial effect of dietary ALA with respect to heart failure risk. Atrial fibrillation and heart failure are frequently comorbid conditions. It seems probable that diets that substantially increase ALA consumption with concurrent decrease in omega-6 PUFAs intake would beneficially affect cardiovascular morbidity and mortality [112]. Calo et al., conducted an open-label, prospective, randomized study in order to assess the efficacy of preoperative and postoperative treatment with omega-3 PUFAs in preventing the occurrence of AF, which is the most coincident complication associated with coronary artery bypass graft surgery [113]. The daily doses of omega-3 PUFAs consisted of two gelatin capsules containing 850 to 882 mg EPA and DHA as ethyl esters with an average ratio of 1:2 EPA/DHA. As a result, postoperative AF was noted in 15.2% (12 of 79) of the patients in the PUFA group compared to 33.3% (27 of 81) of those in the control group. Additionally, the PUFA patients were hospitalized after surgery for significantly fewer days than the controls. This was the first study to demonstrate that individuals supplemented with omega-3 PUFAs presented a decreased risk ratio in cardiac events, like the incidence of postoperative atrial fibrillation [113,114]. ALA’s role either in isoproterenol-treated isolated rat cardiomyocytes or in in vivo rat hearts was studied, and it was demonstrated that an ALA-enriched diet protects the heart against induced fibrosis and hypertrophy [115].

### 2.11. Stroke

Ischemic stroke is considered to be one of the more important reasons of death and prolonged disability among the adults [116,117,118]. Commonly applied and approved stroke therapy involves the use of tissue plasminogen activator (tPA), which, however, is substantially limited by a short temporal window of application [119]. Thus, an important issue in stroke-related research is the search for some alternative therapies that would be safe in the case of long-term prophylactic administration. Although numerous studies have demonstrated that the brain of an adult is able to try to repair itself in reaction to ischemic insults, no safe, efficient therapies that would enhance repair mechanisms and thus prevent neurological deficits induced by stroke have been recognized. Therefore, attention has been paid to neurorestorative therapies that are able to boost cerebral brain repair and concurrently enhance post-ischemia neurological recovery [120,121,122].

Many studies on stroke models have shown that omega-3 fatty acids are able to protect against ischemic brain injury [123,124,125], but the mechanisms of these actions have not been fully explained. It was suggested that this inability to explain the underlying mechanisms behind the protective effect of omega-3 fatty acids could be due to the multiple effects of these acids, i.e., anti-inflammatory activity [125,126], oxidative stress reduction [127], heme oxygenase induction [128], or neurogenesis and oligodendrogenesis potentiation [124]. Palmer et al. [129] also suggested that omega-3s are also able to promote the formation of new blood vessels.

A study conducted by Wang et al. [116] demonstrated that an endogenous post-stroke angiogenesis was induced by omega-3 supplementation, and this measure may be considered to be a potential angiogenic factor enhancing endogenous tissue repair and improving long-term functional recovery after stroke. In turn, Zhang et al. [130] demonstrated in their study that prolonged administration of fish oil caused an increase in cerebral omega-3 level and provided long-term histological and neurological protection against ischemic brain damage. This study also demonstrated that omega-3 PUFAs derived from dietary supplementation can actively promote brain repair due to the possibility of post-stroke brain revascularization as well as enhanced neurogenesis and oligodendrogenesis, not only alleviate ischemic brain injury. 

The study conducted by Mozaffarian et al. [131] revealed that total omega-3 level in plasma was inversely correlated with ischemic stroke risk; however, the authors did not demonstrate any significant effect in the case of hemorrhagic stroke [131]. A meta-analysis of fish consumption relation to stroke conducted by Larsson and Orsini [132] demonstrated that increased fish consumption (three servings per week) was associated with a 6% lower stroke incidence [132]. Also, a meta-analysis of cohort studies performed by He et al. [133] showed an inverse relationship between stroke (especially ischemic one) and fish consumption. However, in another study [134], fish oil supplementation was not related to a decreased stroke incidence. Also, a meta-analysis conducted by Siscovick et al. [135] provided only minor evidence of stroke incidence decrease in patients supplemented with omega-3 PUFAs. Similar conclusions were reported by other meta-analyses [136,137,138].

It can thus be concluded based on the aforementioned evidence that fish consumption is more efficient in stroke prevention compared to omega-3 supplements [139]. Also, a 12-year follow-up study in men demonstrated a 45% lower risk of ischemic stroke, but no change in hemorrhagic stroke incidence, in the case of fish consumption (2–4 servings/week) [140]. Despite the beneficial effects related to fish consumption and stroke incidence reduction demonstrated above, some other studies did not provide such strong evidence [141,142]. The discrepancies observed in different studies conducted on various populations may be due to different patterns and kinds of fish consumed as well as their preparation manners [142,143,144].

## 3. Safety Concerns

Despite abundant evidence on the beneficial activity of omega-3 fatty acids on human health, including cardiovascular diseases, there are also some concerns regarding the safety of their administration. The main concern is related to the possibility of an increased bleeding risk as a result of omega-3s supplementation. According to Bays [145], the involvement of omega-3s in eicosanoid metabolism may be the biochemical cause of possible increased bleeding in the case of increased omega-3 fatty intake. The author points out, that although there is little evidence for an increased risk for clinically significant bleeding incidence with omega-3 fatty acid supplementation, this should be taken into account.

It was suggested that the antithrombotic effect of omega-3s can partially increase the bleeding risk, or even a sudden cardiac death. It was suggested, moreover, that fish oil therapy can cause a slightly higher risk of hemorrhagic stroke, but clinical evidence has not related an increased bleeding with omega-3 fatty acid consumption, even in combination with other agents that potentially increase bleeding (e.g., aspirin) [146]. This was confirmed in the study conducted by Watson et al. [147].

A study concerning the relationship between omega-3 fatty acid index and bleeding in the course of AMI was conducted by Salisbury et al. [148]. The authors suggested that, despite numerous benefits related to CVDs, omega-3 acids can also inhibit platelet aggregation, thus increasing the risk of bleeding. However, following a study on a large, multicenter group of AMI patients, the authors concluded that there is no relationship between the bleeding and omega-3 index, and thus the concerns about bleeding should not prevent the use of omega-3 supplementation in the case of clinical indications.

Another concern related to the safety of omega-3s is the high instability of fish oil and its susceptibility to oxidation, which may contribute to intolerance by patients and an increased risk of toxicity [145,149,150]. Finally, fish consumption may also be related to the risk of poisoning with environmental toxins like mercury, polychlorinated biphenyls, dioxins, or hypervitaminosis due to consumption of fish oils containing high levels of fat-soluble vitamins D and A [145,151,152]. However, these risks can be reduced substantially via purification processes used during fish oil supplements and medical preparations production or supplementation with plant origin omega-3s [145].

## 4. Perspectives

Omega-3 fatty acids have been shown in epidemiological and clinical trials to reduce the incidence of CVD. The daily intake of fish or plant origin oil rich in omega-3 fatty acid corresponding to recommended dosage may prove to be difficult, albeit valuable. Thus, an alternative method of supplementation may be oral omega n3-PUFA therapy, which represents a potentially huge factor of interest and motivation for the pharmaceutical industry. Pharmaceutical and biomedical companies are increasingly interested in the subject of omega-3 fatty acids and their metabolites, and subsequently there has been a substantial amount of research on supplements of omega-3s, particularly those found in seafood and fish oil, and heart disease. Omega-3 fatty acid supplements usually do not have negative side effects. When side effects do occur, they typically consist of minor gastrointestinal symptoms, such as belching, indigestion, or diarrhea. Although the biggest group of omega-3 supplements is based on marine or fish oils, vegetable oils have become increasingly popular and approachable in this matter, not only for vegans and vegetarians but also for people concerned about having a healthy balanced diet. Commonly used alternative dietary supplements based on α-linolenic acid include flaxseed oil and algae oils as a source of DHA. Plant oils (source of ALA) serving as an omega-3 supplements are as potent and effective as fish based oils (containing mainly EPA and DHA fatty acids). However, ALA with only three double bonds is not as susceptible to oxidation as EPA (five bonds) and DHA (six bonds). A substantial part of fish oils is oxidized during the production process, which significantly reduces the pharmacological activity and may confer toxic properties.

## 5. Conclusions

Exogenous polyunsaturated fatty acids deserve special attention, given the importance of their physiological functions, determined by the limited capacity of fatty acid desaturation by human tissues. As is clear from the above facts, omega-3 fatty acids, particularly alpha-linolenic acid (the pharmacologically active precursor of EPA and DHA), have a broad spectrum of anti-inflammatory and cardio-protective activities. There is, however, still need for further research into the function of ALA as an independent nutrient. In conclusion, the beneficial effects of omega-3 fatty acids and their esters have been previously documented in terms of both primary and secondary prevention of cardiovascular system disorders. It has to be highlighted, however, that the achievement of optimal performance requires an appropriate quantitative composition and the proper proportions of delivered fatty acids. A healthy balanced diet, especially when combined with regular physical activity and smart supplementation of omega-3 fatty acids, has been reported as being effective in preventing cardiovascular events, cardiac death, and coronary events, especially in persons with high cardiovascular risk. 

## Abbreviations

AA	arachidonic acid (20:4, n-6)
AF	atrial fibrillation
ALA	alpha-/α-linolenic acid (18:3, n-3)
AMI	acute myocardial infarction
CAD	coronary artery disease
CVD	cardiovascular disease
DHA	docosahexaenoic acid (22:6, n-3)
EFA	essential fatty acid
EPA	eicosapentaenoic acid (20:5, n-3)
GLA	gamma-/γ-linolenic acid (18:3, n-6)
HDL	high-density lipoprotein
IHD	ischemic heart disease
LA	linoleic acid (18:2, n-6)
LDL	low-density lipoprotein
MI	myocardial infarction
MUFA	monounsaturated fatty acid
PUFA	polyunsaturated fatty acid
TGs	triglycerides
VLDL	very low-density lipoprotein
WHF	World Health Federation
WHO	World Health Organization
